# 

**Published:** 1891-05-30

**Authors:** 


					? INFANTS t n C. '?!
No. 244. Vol. X.] ,k Saturday,
May 80tk, 1391.
)NE Pexvv.

				

